# Microplastics in gentoo penguins from the Antarctic region

**DOI:** 10.1038/s41598-019-50621-2

**Published:** 2019-10-02

**Authors:** Filipa Bessa, Norman Ratcliffe, Vanessa Otero, Paula Sobral, João C. Marques, Claire M. Waluda, Phil N. Trathan, José C. Xavier

**Affiliations:** 1 MARE – Marine and Environmental Sciences Centre, Departamento das Ciências da Vida, Faculdade de Ciências e Tecnologia, Universidade de Coimbra, 3000-456, Coimbra, Portugal, 3004-517 Coimbra, Portugal; 2 British Antarctic Survey, Natural Environment Research Council, High Cross, Madingley Road, Cambridge, CB3 0ET UK; 3 Department of Conservation and Restoration and LAQV-REQUIMTE, Faculdade de Ciências e Tecnologia da Universidade Nova de Lisboa, 2829-516 Monte de Caparica, Portugal; 4 MARE-NOVA – Faculdade de Ciências e Tecnologia, Universidade NOVA de Lisboa, Campus da Caparica, 2829-516 Caparica, Portugal

**Keywords:** Ecology, Ecology, Ecology, Ecology

## Abstract

There is growing evidence that microplastic pollution (<5 mm in size) is now present in virtually all marine ecosystems, even in remote areas, such as the Arctic and the Antarctic. Microplastics have been found in water and sediments of the Antarctic but little is known of their ingestion by higher predators and mechanisms of their entry into Antarctic marine food webs. The goal of this study was to assess the occurrence of microplastics in a top predator, the gentoo penguin *Pygoscelis papua* from the Antarctic region (Bird Island, South Georgia and Signy Island, South Orkney Islands) and hence assess the potential for microplastic transfer through Antarctic marine food webs. To achieve this, the presence of microplastics in scats (as a proof of ingestion) was investigated to assess the viability of a non-invasive approach for microplastic analyses in Antarctic penguins. A total of 80 penguin scats were collected and any microplastics they contained were extracted. A total of 20% of penguin scats from both islands contained microplastics, consisting mainly of fibers and fragments with different sizes and polymer composition (mean abundance of microplastics: 0.23 ± 0.53 items individual^−1^ scat, comprising seven different polymers), which were lower values than those found for seabirds in other regions worldwide. No significant differences in microplastic numbers in penguin scats between the two regions were detected. These data highlight the need for further assessment of the levels of microplastics in this sensitive region of the planet, specifically studies on temporal trends and potential effects on penguins and other organisms in the Antarctic marine food web.

## Introduction

Plastic pollution (whether at the macro or the micro scale) is one of today’s environmental issues of highest concern among scientists, policy makers and the general public^1–4^. One aspect of plastic pollution that has received increasing attention in recent years is that of microplastics, defined as any plastic particles smaller than 5 mm, which are either manufactured as small particles or originated from the fragmentation of larger plastic items^5,6^. These particles may be subjected to a range of complex processes in the environment (weathering, embrittlement, aggregation), producing a variety of particles with different characteristics (shape, size and density)^2,7^. Additionally, they can leach out chemical additives and adsorb organic chemicals that are found in the environment, and some such compounds can bioaccumulate, posing a potential detriment to species and ecosystems^4,8^.

Microplastics are present in most habitats including rivers, lakes, oceans, soil and air^9–11^ and are bioavailable to ingestion by a variety of aquatic biota (from zooplankton to megafauna)^12–16^. Despite their widespread occurrence, their effects on wild organisms have yet to be quantified^4,8^. These particles have been detected in remote areas such as the Arctic and the Antarctic regions^17,18^. These polar ecosystems have recently received heightened attention regarding microplastic pollution, and there are several studies that have reported evidence of these particles in Arctic surface waters^19,20^, in high concentrations in the sea ice across the Arctic Ocean^18^ and in benthic organisms^21^.

Less is known about the occurrence of microplastic in the Southern Ocean^17^, where they have only been detected in seawater^22,23^ and sediments^24,25^. However, it has been shown that Antarctic krill *Euphausia superba* can ingest microplastics in laboratory experiments^26^, but less is known regarding the levels of microplastics in wild Antarctic marine organisms (but see the reports on ingestion of plastic debris of larger sizes by petrels breeding in Antarctica^27^ and fur seals^28^.

Despite its remoteness, the Antarctic environment may be subject to local plastic pollution sources from fishing activities, tourism or research stations^17^. Microplastics could therefore reach Antarctica via three distinct routes: from local human activities, from outside Antarctica in atmosphere/ocean circulation or bio-transport by organisms that migrate to Antarctica from waters further north and induced by storm-driven dispersal and global warming^29^. However, the data available regarding these processes in the Antarctic are still so limited that the Scientific Committee on Antarctic Research has recently created an action group on plastic pollution (https://www.scar.org/science/plastic/about/).

Within Antarctic marine organisms, seabirds are considered as one of the most convenient bioindicators of environmental change and have been also proposed as indicators for plastic pollution in many marine environments^30,31^. Antarctic penguins, such as gentoo penguins *Pygoscelis papua*, have been considered a standard organism for monitoring contamination in Antarctic marine ecosystems^32,33^. Gentoo penguins are also an appropriate species in this context due to their limited movements outside their home range throughout the year, as shown by tracking data, diet and genetic studies^34–38^, which exclude the possibility of ingesting plastic debris from waters outside Antarctica. The aim of this study was thus to assess the occurrence of microplastics in an Antarctic marine predator in the wild, the gentoo penguin, (using their scats as proxy of ingestion), to identify and characterize the microplastics found, and discuss their likely source.

## Results

**Figure 1:**
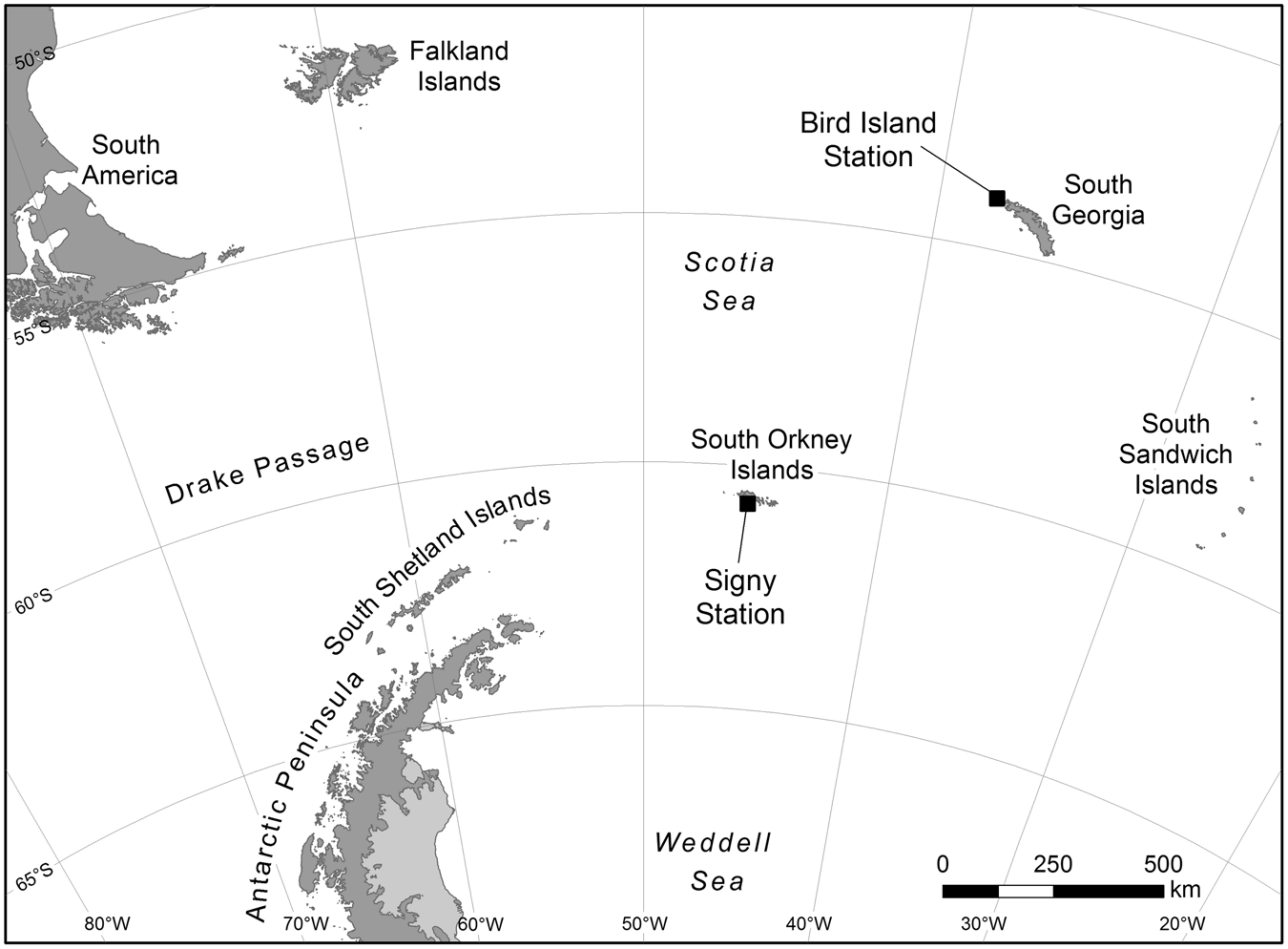
Map of location of the sampling islands (Bird Island and Signy Island) in the Scotia Sea.

A total of 80 scats were collected (50 scats at Bird Island and 30 scats at Signy Island, Fig. 1). All isolated particles (suspected microplastics) were characterised, but only those confirmed by micro-Fourier Transform Infrared Spectroscopy (µ-FTIR) as synthetic/semi-synthetic were considered in the total counts. In addition, we identified two particles of cellulose and five other particles as natural compounds; these were excluded from the analyses. No microplastics were detected in any of the blank replicates.

No significant differences between the two islands were found regarding the number of scats having microplastics (PERMANOVA test: pseudo-F = 0.0501 and p (MC) = 0.8283) and 20% of scats were found with microplastics in Bird Island (13 microplastics in 10/50 scats) and 20% of scats with microplastics were recorded in Signy Island (6 microplastics in 6/30 scats).

**Figure 2:**
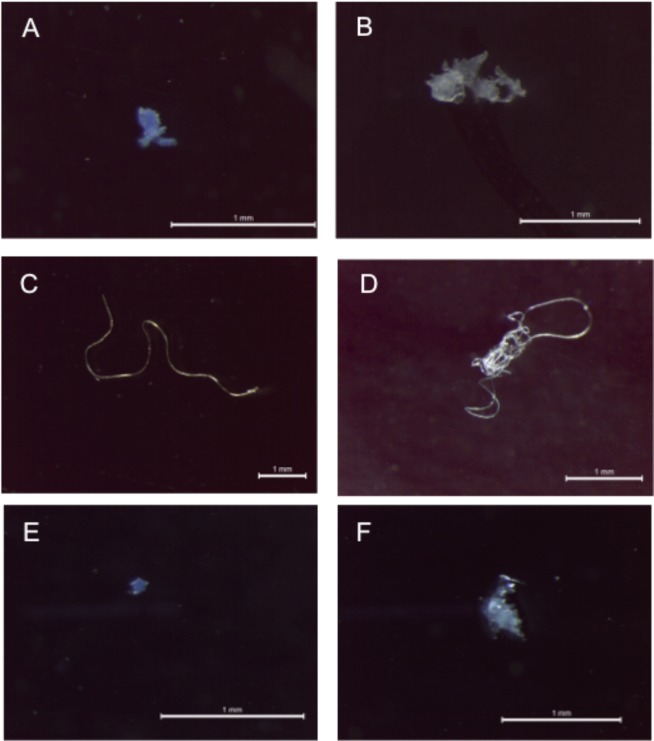
Example of microplastics isolated from penguin scats and analysed using *u*-FTIR. (**A**) Blue polyester fragment; (**B**) Transparent polyethylene film; (**C**) Transparent polyacrylonitrile fiber (**D**) Mixed polyester fibers; (**E**) Blue polyacrylate fragment and (**F**) Transparent Rayon film. Scale Bars = 1 mm. (For interpretation of the references to colour in this figure legend, the reader is referred to the Web version of this article).

In total, 19 microplastics were identified from the scats (0.23 ± 0.53 items.scat^−1^) and microfibers were the most abundant shape in the scats from both islands (58%), followed by fragments (26%) and films (16%) of different colours and sizes (Table 1 and Fig. 2). Of the 16 samples with microplastics, there was on average 1.19 ± 0.54 items.scat^−1^: range 1–3 particles) with different mean values from both islands (Bird Island: 1.3 ± 0.67 items.scat^−1^ and Signy Island: 1 items.scat^−1^). Microplastics measured on average 1266 ± 1378 µm with a minimum length of 76 µm and a maximum of 4945 µm (Table 1). Mean fiber length was 1889 ± 1448 µm (range 408–4945 µm), while fragments and films measured on average 312 ± 229 µm (range 76–738 µm).

**Table 1:** Identification of the synthetic particles found in penguin scats collected at the Southern Ocean. For the infrared characteristic band of the microplastic samples please see supplementary material.

Sampling site	Polymer Type	Shape	Colour	Size (µm)
Bird Island	Unidentified*	Fragment	Green	76
	Polyacrylonitrile	Fiber	Transparent	2204
	Polyester	Fragment	Red	173
	Polyester	Fiber	Red	570
	Polyester	Fragment	Blue	306
	Polyester	Fiber	Blue	957
	Polyester	Fiber	Blue	408
	Polyester	Fiber	Blue	2230
	Polyester	Fiber	Black	1908
	Polyester	Fiber	Transparent	3980
	Polyester	Fiber	Transparent	495
	Polypropylene	Film	Black	738
	Polyethylene	Film	Transparent	486
Signy Island	Cellulose (Rayon)	Fragment	Blue	159
	Cellulose (Rayon)	Film	Transparent	446
	Cellulose (Rayon)	Fiber	Transparent	1366
	Polyacrylate	Fragment	Blue	118
	Polyester	Fiber	Transparent	1717
	Polyester	Fiber	Blue	4945

*Similar to a poly(ethylacrylate:st:acrylamide) see Table 1S–G.

Seven different polymer types were identified in penguin scats from both locations (Table 1). Polyester accounted for the largest proportion (60%) being mainly microfibers, followed by fibers of the semi-synthetic cellulose polymer Rayon (15%, only detected in Signy Island), whereas the other particles (fragments and films) were identified as single polymers (polyacrylonitrile, polypropylene, polyethylene and polyacrylate, Table 1 and Supplementary material). One particle was classified as “unidentified synthetic” despite the clear similarity with a synthetic poly(ethylacrylate:st:acrylamide), however, additional bands are observed that may be due to degradation of the polymer or to the presence of another compound (see Fig. 1S–G and Table 1S). Nevertheless, due to its anthropogenic origin this was considered in the total counts of microplastics. In addition, the composition of the polymers varied in the two islands with 3 polymer types being recorded in Signy Island (Rayon, polyacrylate and polyester), while a higher diversity was recorded for microplastics found in Bird Island (polyacrylamide, polyacrylonitrile, polyester, polypropylene and polyethylene, Table 1).

## Discussion

To our knowledge, this is the first study to show that microplastics are present in the gastrointestinal tract of penguins feeding in Antarctic waters and therefore potentially widespread in the Antarctic marine food web. Microplastics have probably been ingested and taken up by gentoo penguins either through direct ingestion (e.g. due to misidentification of microplastics for food), indirect ingestion via contaminated prey (e.g. Antarctic krill is capable of ingesting microplastics in laboratory experiments^26^, so it is likely that it may also ingest microplastics in the wild), and/or incidental ingestion via contaminated water/sediments (i.e. microplastics in Antarctic sediments have been noted^23–25^ or while preening their feathers). According to several studies, the diet of gentoo penguins usually includes a high percentage of Antarctic krill^37–40^, so the observed contamination of gentoo scats implies that other Antarctic krill dependent species may also be susceptible of being contaminated with microplastics and need to be addressed in future research. Antarctic krill play a key role in Antarctic marine ecosystems^41^, in which the majority of primary productivity is passed to secondary consumers through a single main grazing species (i.e. Antarctic krill)^42^. Thus, microplastics may be passed among numerous organisms within the Antarctic marine food web.

In this study, 80 scats from gentoo penguins were analysed from two islands from the Southern Ocean (Bird Island and Signy Island), with no significant differences in the amount of contaminated scats between sites. However, there were dissimilarities in the polymeric composition of the particles. These differences in polymer composition of the extracted particles can be attributed to potential different point sources of plastic pollution in these regions. Both islands do have research stations and fisheries operating in their waters^43,44^, but Bird Island is located considerably closer to the Antarctic Polar Front and potentially more influenced by pollution coming from the Antarctic Circumpolar Current and from the north, whereas Signy Island is located much closer to the Antarctic continent, further away from potential northern sources of pollution less influenced by Antarctic Circumpolar current^45,46^. However, to categorically attribute cause, or source, would require background data on the prevalence of different compound types with latitude in order to make any deductions with confidence.

At both our study sites, microfibers were the main category of microplastics recorded, which agrees with a general pattern reported in the marine environment^17,19^ and in similar studies performed during ingestion studies for marine species from other habitats^12,14,16^. The potential sources of these microfibers in other marine environments has been attributed to the generation of fibers during clothes washing^9^, or also from the fragmentation of nets and ropes from fisheries discarded in the oceans^10^. Microplastic fragments of different shapes and colours will be difficult to trace to source based simply on polymer composition. Moreover, currently available information on the distribution of microplastics is not sufficient to identify potential sources of microplastic pollution and hence now warrants further assessment^47,48^. In the southwest Atlantic, additional point sources from anthropogenic activities can be diverse including direct sources via disposal, inadequate management of waste produced by ships or from touristic activitites^17^. Narrowing down the source of microplastic will only become feasible with background data on prevalence of different compound types.

Existing data on the concentration of a range of other persistent contaminants in air, snow and marine organisms in the Antarctic environment has already demonstrated that these pollutants are transported from other continents in the Southern Ocean^49^, and this could be also the case with plastic debris. For this reason, it is also important to point out the potential contribution of the atmospheric fallout^48,50^ as input of microplastics, especially microfibers in this area.

The proportion of scats containing microplastic was relatively low, when compared with results for other seabirds, or other top predators, from other environments^31,51^, which is consistent with the limited number of studies that reported similar relatively low values of these anthropogenic particles in water and sediments in this region^17^.

Overall, our data confirm that microplastics are present in penguins from the Antarctic and sub-Antarctic region. We have shown that penguin scat analysis is a suitable approach for assessing anthropogenic changes in Antarctica regarding the presence and distribution of microplastics since it is a non-invasive method and it can be considered a suitable first approach for areas or species that are understudied. However, there are also limitations, since microplastics recovered from scat samples represent those particles that were ingested and excreted and caution is recommended when assessing potential effects of those plastics passing through the digestive systems of these species^31^. Further work is needed to evaluate the fate and transport of microplastics in the Antarctic ecosystem and their impacts on key species. Indeed, Antarctica may still be relatively isolated from microplastic pollution, but not completely so; this data refers to 2009 and since it is known that plastic pollution has increased and is expected to increase in the near future^52^, the situation can change rapidly. Surface currents connect Antarctica with the rest of the planet and may allow the entrance of microplastics^17,29,53^. In contrast, local sources of microplastics are relatively small as the number of research stations, tourist vessels and fishing vessels is still small, making Antarctica very sparsely populated compared with elsewhere on the planet. Nevertheless, some authors^17^ estimate that about 25.5 billion fibers of synthetic origin are potentially entering the Southern Ocean per decade from clothes laundering, making a substantial contribution to the local levels of microplastics. A much wider investigation would be needed to reveal the full spectrum of microplastic pollution in Antarctica.

## Methods

Gentoo penguin scats were collected from colonies at Bird Island, South Georgia (54°S, 38°W) and Signy Island, South Orkney Islands (60°S, 45°W), during the austral summer of 2009 (Fig. 1). Samples were collected by hand using nitrile gloves and placed into sterile collection plastic bags. Scats were randomly collected across sites to avoid the possibility of collecting scats from the same individual. Scats were collected from the ground immediately after defecation following the procedures described in^37^. Each scat was frozen (−20 °C) for further analyses in the laboratory. Each scat was then defrosted and transferred to a clean glass beaker (250 ml) and 100 ml of potassium hydroxide solution (KOH, 10%) was added to digest the organic material. After 24 h of digestion at 40 °C, the floating phase was vacuum filtered through a 1.2 µm glass microfibre filter. The residue was transferred to a new glass beaker and hydrogen peroxide (H_2_O_2_, 10%, 24 h) was added to increase the recovery of potential particles trapped in the residue and the filtration was repeated. Each filter was placed in a Petri dish and placed to dry in an oven at 40 °C. Three identical blanks in total with milliQ water were set up, using the same procedures to account for background contamination. The filter papers were then examined under a stereomicroscope LEICA M80 (Microsystems GmbH, Wetzlar, Germany), to identify potential microplastics. Particles exhibiting the appearance of microplastics/anthropogenic particles were kept on filters, photographed using the image analysis system IC80 HD Camera with Leica Application Suite (LAS) software and placed between two microscopic slides, until further analysis. The particles were categorized according to their shape and colour and their largest cross-section (size) was measured. The lower limit of resolution and detection of microplastics was 60 µm. The chemical composition of all particles collected was determined by micro-Fourier Transform Infrared Spectroscopy (*µ*-FTIR) and infrared spectra were acquired in a Nicolet® Nexus spectrophotometer coupled to a Continuum microscope (15× objective) with a MCT-A detector cooled by liquid nitrogen, following the technical procedures described in^14^. The polymer identification was based on spectral absorption bands and confirmed by comparison with a spectral library database. To avoid potential airborne contamination, all liquids used were previously filtered through a 1.2 µm glass microfibre filter and glass containers rinsed with ultrapure water before reuse. Moreover, the entire process of analyses of microplastics (extraction and identification) was conducted by only one person in a closed and restricted access laboratory. Laboratory work was carried out using a cotton lab coat and nitrile gloves.

Microplastic concentrations were compared between sites using a permutational multivariate analysis of variance (PERMANOVA^54^), as data were not normally distributed (Kolmogorov-Smirnov: *p* < 0.05) and not homoscedastic (Levene’s test *p* < 0.05). All statistical analyses were performed using PRIMER v.6 and its add-on package PERMANOVA+^54^.

## Supplementary information

Suplementary material
