# Supplementary material for: Microplastics in gentoo penguins from the Antarctic region
Source: Sci Rep. 2019 Oct 2;9:14191. doi: 10.1038/s41598-019-50621-2 (PMC6775258; doi:10.1038/s41598-019-50621-2)
Supplement: Supplementary file 1 — Suplementary material [file 41598_2019_50621_MOESM1_ESM.pdf]

Microplastics in gentoo penguins from the Antarctic region

Filipa Bessa[1 *], Norman Ratcliffe [1,2], Vanessa Otero [3], Paula Sobral [4], João C. Marques [1], Claire M. Waluda[2], Phil N. Trathan[2], José C. Xavier [1,2]

[1] MARE – Marine and Environmental Sciences Centre, Faculdade de Ciências e Tecnologia, Universidade de Coimbra, 3004-517 Coimbra, Portugal

[2] British Antarctic Survey, Natural Environment Research Council, High Cross, Madingley Road, Cambridge, CB3 0ET, UK

[3] Department of Conservation and Restoration and LAQV-REQUIMTE, Faculdade de Ciências e Tecnologia da Universidade Nova de Lisboa, 2829-516 Monte de Caparica, Portugal

[4] MARE-NOVA – Faculdade de Ciências e Tecnologia, Universidade NOVA de Lisboa, Campus da Caparica, 2829-516 Caparica, Portugal

Table 1S – Infrared characteristic bands (cm^{-1}) of the identified polymers. Adapted from: Hummel, 2002. (ν – stretching; δ_{as} – asymmetric bending; δ_s – symmetric bending; intensity band: vs – very strong; s – strong; m – medium; w – weak).

Polymer	Characteristic band (cm^{-1})	Assignment
Polyethylene	2918 vs, 2850 s	$\nu_{as}(CH_2)$, $\nu_s(CH_2)$
	1472 w	$\delta_{as}(CH_2)$
Polypropylene	2960 vs, 2877 m	$\nu_{as}(CH_3)$, $\nu_s(CH_3)$
	2918 s, 2838 m	$\nu_{as}(CH_2)$, $\nu_s(CH_2)$
	1460 m	$\delta_{as}(CH_3)$
	1377 m	$\delta_s(CH_3)$
Semi-synthetic cellulose Rayon	3650-3000 s	ν(OH)
	2990-2820 w	ν(CH)
	1425, 1372, 1320 m	δ(CH)
	1200-1000 vs	δ(C-OH) + δ(C-C) + ν(C-O-C) + ν(C-OH)
Polyester	2966 w	ν(CH)
	1720 vs	ν(C=O)
	1408 m	δ(ring)
	1340 m	δ(CH)
	1261/1246 s, 1117/1102 s	ν(COC)
	1018 m, 728 m	δ(CH ring)
Polyacrylonitrile	2935 m, 2870 m	$\nu_{as}(CH_2)$, $\nu_s(CH_2)$
	2243 vs	ν(CN)
	1731 s	ν(C=O)
	1451 s	$\delta(CH_2)$
Polyacrylate	2960-2870 w	ν(CH)
	1731 vs	ν(C=O)
	1270 w, 1242 w, 1150 s	ν(COC)

Fig. 1S – Infrared spectra of the microplastic samples (below) and the respective polymer (synthetic and semi-synthetic) reference spectra (top) identified as: A) Polyethylene; B) Polypropylene; C) Rayon (Semi-synthetic cellulose); D) Polyester; E) Polyacrylonitrile; F) Polyacrylate and G) similar to poly(ethylacrylate:st:acrylamide).

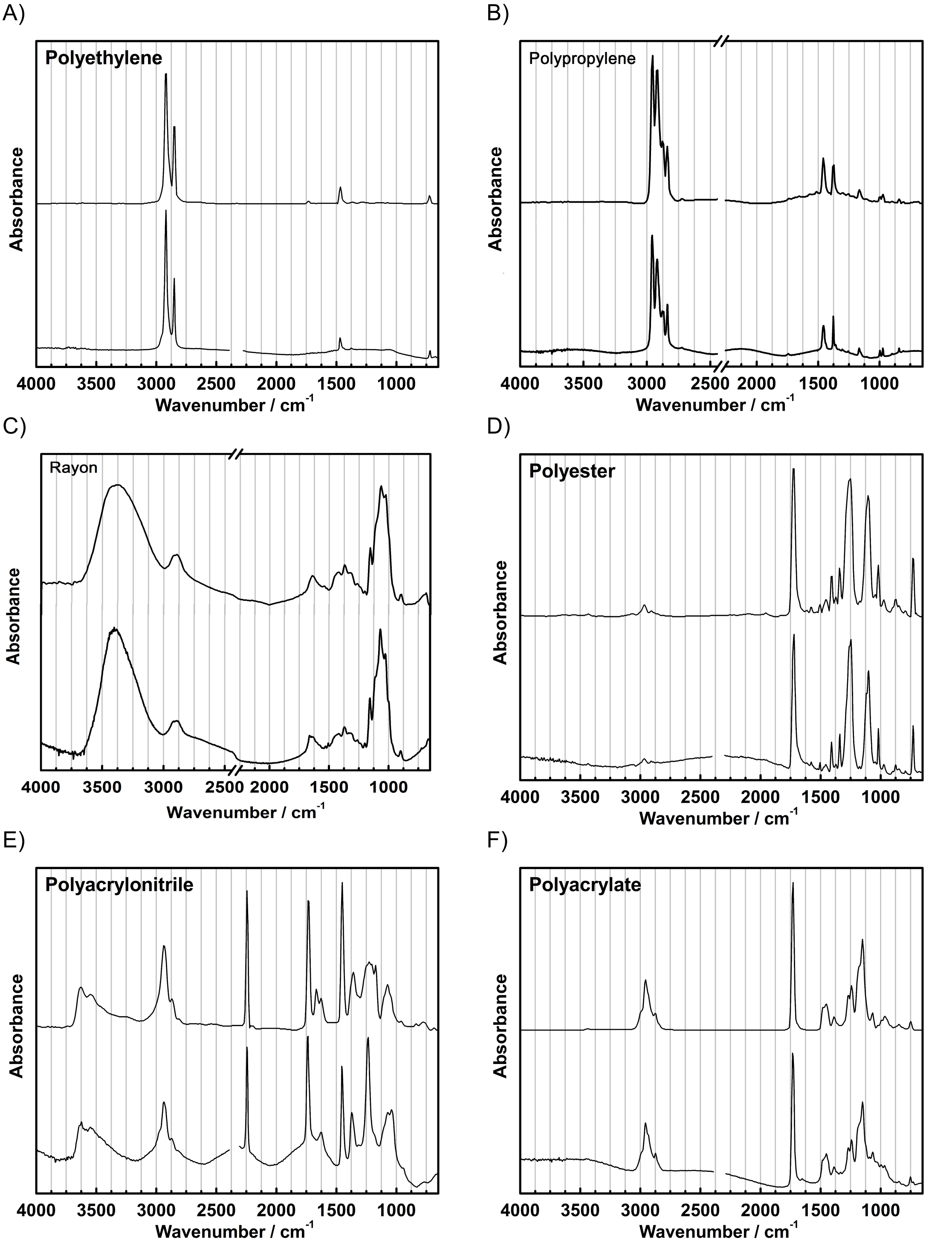

G)

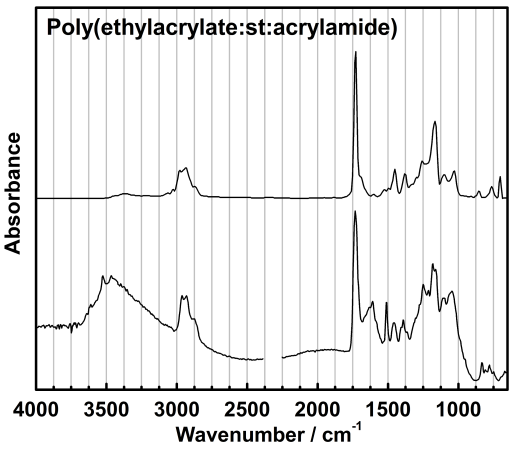
Poly(ethylacrylate:st:acrylamide)
Absorbance
4000
3500
3000
2500
2000
1500
1000
Wavenumber / cm-1
